# Supplementary material for: Trends in Botulinum Toxin Use among Patients with Multiple Sclerosis: A Population-Based Study
Source: Toxins (Basel). 2023 Apr 12;15(4):280. doi: 10.3390/toxins15040280 (PMC10142089; doi:10.3390/toxins15040280)
Supplement: Supplementary file 1 [file toxins-15-00280-s001.zip › toxins-2271170-supplementary.pdf]

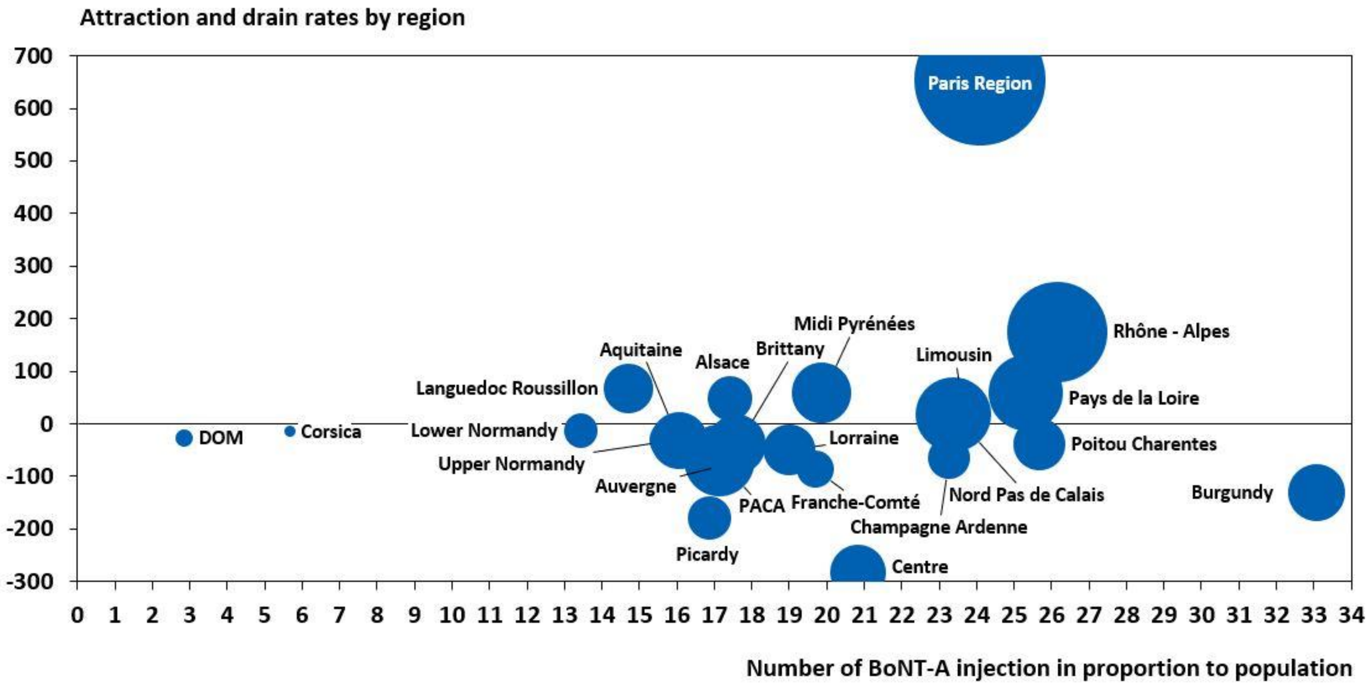

Figure S1. Distribution of intradetrusor botulinum toxin type A (BoNT-A) injections per region. Urban regions such as Paris Region and Rhône-Alpes (southeast region of France) attract patients, whereas in rural regions such as Centre and Picardy, there is a drain of patients needing to have intradetrusor BoNT-A injections. Abbreviations: DOM, overseas departments; PACA, Provence-Alpes-Côte d'Azur.
